# Spatiotemporal Distributions of Sheep and Goat Pox Disease Outbreaks in the Period 2013–2019 in Eastern Amhara Region, Ethiopia

**DOI:** 10.1155/2021/6629510

**Published:** 2021-01-05

**Authors:** Sileshi Aregahagn, Belege Tadesse, Bethelihem Tegegne, Yalelet Worku, Seid Mohammed

**Affiliations:** ^1^Wollo University, School of Veterinary Medicine, P.O. Box. 1145, Dessie, Ethiopia; ^2^Kombolcha College of Animal Health and Agriculture, Kombolcha, Ethiopia; ^3^Kombolcha Regional Veterinary Laboratory, P.O. Box. 09, Kombolcha, Ethiopia

## Abstract

Sheep and goat pox is highly contagious viral infection of sheep and goats caused by the genus Capripox virus. Clinically, the disease is characterized by fever, macules developing into papules, and necrotic lesions in the skin and nodular lesions in internal organs. In Ethiopia, there are seroprevalence epidemiological studies on the disease. However, the spatiotemporal clustering of sheep and goat pox incidence has not been investigated. A retrospective study design using the outbreak reported data from Kombolcha Regional Laboratory for the years from September 2013 to December 2019 was performed to determine the temporal and spatial distribution of sheep and goat pox outbreaks. A total of 663 sheep and goat pox disease outbreaks were reported in all major parts of Eastern Amhara region between 2013 and 2019. In this period, sheep and goat pox was reported in all administrative zones of Eastern Amhara region (*n* = 5). The average incidence of sheep and goat pox outbreaks at the district level was 8.61 per 7 years. The incidence differed between areas, being the lowest in hot dry month and highest in warm and cold moist months. Sheep and goat pox outbreaks generally have a peak in November followed by August and a low in May. There is a significant difference in the occurrence of sheep and goat pox disease outbreaks between months and years (*p* < 0.001). The forecast for the period 2020–2026 revealed that a high number of sheep and goat pox disease outbreaks will occur than the previous one. Therefore, all stakeholders should work cooperatively to combat this disease occurrence, and there should be capacity development for participatory disease search, risk analysis, laboratory diagnosis, and information management in order to respond properly to outbreak of sheep and goat pox disease; thereby, it enhances the prevention and control the disease.

## 1. Introduction

Sheep and goat pox (SGP) is a highly contagious viral infection of sheep and goats [1] caused by the genus Capripox virus, one of the six genera of poxviruses of vertebrates [2]. Sheep pox and goat pox were regarded as unlike diseases in the past [3]. Goat pox was reported at first in 1879 from Norway [4]. As indicated by Blanc et al. [5], it was recorded during the First World War in Macedonia and became enzootic during 1926 with a mortality rate of 15%. The causative virus of sheep pox is antigenically and genetically closely related to goat pox virus and lumpy skin disease virus. Sheep pox and goat pox are characterized by fever, macules developing into papules and necrotic lesions in the skin, and nodular lesions in internal organs, secondary infections, and death in susceptible stock [6]. This disease causes a morbidity of 75–100% and case fatality, depending on the virulence of the virus between 10% and 85% [7].

This disease is spread by the direct contact with infectious animals and indirect contact with contaminated objects; for example, the virus could survive for many years in dried scabs at ambient temperatures and for 2 months on wool [8]. Most studies showed that the majority of sheep and goat pox outbreaks occur during the winter and spring months [2, 9].

The fatality of the disease varies between different age groups of the animals. For example, in lambs, the disease is fatal resulting in death. The diseases are characterized by symptoms of fever and paralysis and an eruption in the form of red spots that appear on the membranes of the eyes and nose and on the wool-free parts of the skin. In older sheep, the disease begins with serious ill-health signs, notably a high temperature and suppressed appetite [10]. Infected pregnant ewes often abort.

The diagnosis of sheep pox is usually based on highly characteristic clinical signs [11], virus isolation, the virus neutralisation test (VNT) [8], serological assays (e.g., ELISA) [12], and PCR assays [13].

The sheep pox and goat pox diseases are among the most important diseases of sheep and goats in Ethiopia next to peste des petits ruminants (PPR) and contagious caprine pleuropneumonia (CCPP) [14, 15]. Sheep pox and goat pox are found in all regions of Ethiopia; in 2007/2008, the Animal and Plant Health Regulatory Directorate received 893 sheep and goat pox outbreak reports from all regions except Gambella, Harari, and Diredawa. From these outbreaks, a total of 57,638 sheep and goats contracted the disease. Out of the 57,638 sick sheep and goats, 6,401 animals died. As a control measure, vaccination was used throughout the country even if animal owners did not regularly vaccinate their animals, which becomes a reason for outbreaks. The diseases reporting rate in Ethiopia is low only about 35–40%. The actual figures in terms of affected, vaccinated, and dead animals are, therefore, expected to be higher than the reported figures [16].

In Ethiopia, inadequate works have been performed on seroprevalence, risk factors, and distribution of sheep and goat pox virus except in selected areas of Afar region and Western Amhara region [17, 18]. In addition, limited works have been performed on geographical and temporal distribution of SGP diseases in Ethiopia in general and Amhara region, in particular. Evaluation and a better understanding of the spatial and temporal distributions of SGP disease outbreaks are preconditions for directing successful surveillance and disease control efforts in the study area. Therefore, the objective of this study was to identify the spatial and temporal distribution of SGP outbreaks in Eastern Amhara region based on data reported to Kombolcha Regional Veterinary Laboratory during the period from September 2013 to December 2019.

## 2. Materials and Methods

### 2.1. Description of the Study Area

**Figure 1:**
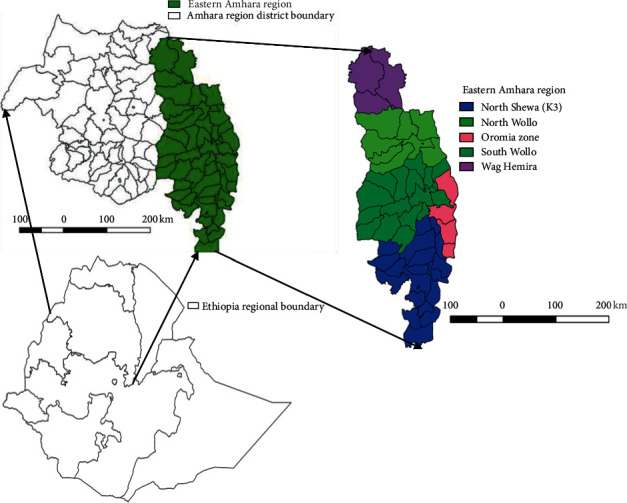
Map of the study zones (produced by using QGIS 2.18.28).

The study was conducted in all Eastern Amhara zones (Wag Hemira, North Wollo, South Wollo, North Shewa, and Oromia). These administrative zones are divided into a total of 77 districts. The study area is located in the northeastern part of Ethiopia between 8°55′ and 13°25′ North latitude and 38°15′ and 40°25′ East longitude (Figure 1). The study area has diverse agroclimatic conditions, ranging from hot lowlands to cold highlands. Areas less than 1500 meter above sea level (m.a.s.l) will be considered as lowland, areas ranging 1500–2500 m.a.s.l will be considered as midland, and areas greater than 2500 m.a.s.l will be considered as highland [19]. The major livelihoods of the people within the study zones is a mixed crop-livestock system, in which small ruminants have a major roll as cash income and as food. In all the study zones in the study regions, there is free animal marketing (owners move their animals for sale freely) which favors disease transmission. There is a vaccination program against sheep and goat pox diseases within the study zones as a control option, but sometimes, some kebeles and districts are irregularly addressed and they fall under an outbreak.

### 2.2. Study Population

The total sheep and goat population in the study zones are given in Table 1. Sheep and goats that were kept under the extensive and semiintensive farming system of all breeds present in the study area were considered in the study. The breeds include indigenous breeds of sheep, Awassi-indigenous crosses, and indigenous breed goats. Sheep and goats are mainly kept in the study area for family diet and cash income.

### 2.3. Study Design

A retrospective study design was used to assess the temporal and spatial distribution of sheep pox and goat pox disease outbreaks in the study zones of Eastern Amhara region.

### 2.4. Outbreak Data Source

Sheep and goat pox diseases are among OIE-listed viral diseases, which require reporting of all occurrences to the national veterinary authority of each OIE member country and internationally to the World Organization for Animal Health. SGP outbreak data for the period September 2013–December 2019 were obtained from the Epidemiology Unit of Kombolcha Regional Veterinary Laboratory. The records included information such as districts, zones, species affected, index date, number of cases, number of outbreaks, number of deaths, number of vaccine given, and number of animals at risk. For this study, an outbreak was defined as one or more sheep or goats showing SGP signs in a district and confirmation of the outbreak by the regional laboratory. Therefore, the SGP outbreak incidence was computed at the district (*n* = 77) level using the seven years (September 2013–December 2019) outbreak data. During data collection, consent was taken with the Epidemiology Unit of Kombolcha Regional Veterinary Laboratory to use the data for this study.

### 2.5. Data Management and Analysis

Microsoft Excel spreadsheets (Microsoft Corporation) were used to manage the data and draw graphs. Descriptive methods were used to calculate outbreak incidence. The mean SGP outbreak incidence was calculated by summing all reported SGP outbreaks over the study period in the region divided by the total number of districts and number of years (district years). The spatial distribution of SGP outbreaks over the study period was drawn by administrative zones using QGIS version 2.18.28 software. The number of SGP outbreaks reported in the seven-year study period was graphed to visualize the temporal trends of the disease. The graph was inspected for the presence of seasonality or long-term trend. The long-term trend of SGP outbreaks was verified by linear regression in STATA version 14 by taking the number of SGP outbreaks as outcome variable and years of the outbreaks as predictor variable.

## 3. Result

### 3.1. Temporal Distribution of SGP Disease Outbreaks in Eastern Amhara Region

From September 2013 to December 2019, 663 clinically diagnosed and laboratory confirmed sheep and goat pox outbreaks were reported to Kombolcha Regional Laboratory from Eastern Amhara region. In the period from September 2013 to December 2019, high number of SGP outbreaks were reported in 2015 (*n* = 189 outbreaks), 2018 (*n* = 135), and 2019 (*n* = 99), while the lowest number of outbreaks were reported in 2014 (*n* = 15). There is a significant difference in the occurrence of SGP outbreaks between years (*p* < 0.001) (Table 2). The predicted number of SGP outbreaks for the years 2020–2026 shows that it will increase to 215 in 2026 if best control measure is not implemented (Table 2).

The seasonality in the numbers of outbreaks is obvious, which tend to be higher in the months starting from the end of summer (August) to dry cold season (spring) compared to other seasons. The highest number of outbreaks was reported in the month of November (*n* = 105 across all years), which accounted for 15.84% of all reported outbreaks and the lowest in May (*n* = 27), accounting for 4.1% of all reported outbreaks. There is a significant difference in the occurrence of SGP outbreaks between months (*p* < 0.001) (Table 3). In general, the number of SGP disease outbreaks was above average for the months August–November and below average for December–July (Table 3). On average, 55.25 SGP disease outbreaks were reported in each month from September 2013 to December 2019.

**Figure 2:**
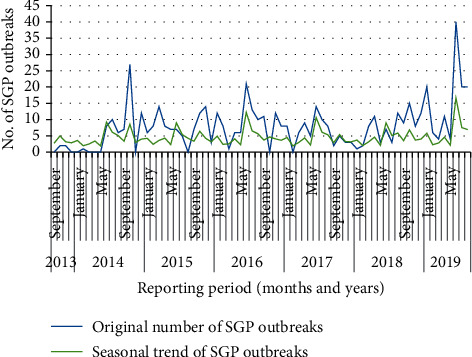
Monthly outbreak and trend of SGP outbreak from 2013 to 2019.

The monthly distribution of SGP outbreaks and seasonal trend is shown in Figure 2. The seasonal trend shows that the highest numbers of outbreaks were report during July 2019.

### 3.2. Spatial Distribution and Incidence of SGP Disease Outbreaks

**Figure 3:**
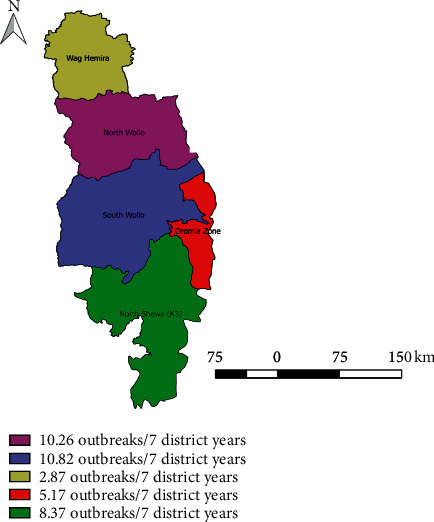
Incidence of sheep and goat pox disease outbreaks in Eastern Amhara zones per 7 district years.

During the period from September 2013 to December 2019, SGP disease has been reported from all administrative zones (*n* = 5) of Eastern Amhara region. All administrative zones in Eastern Amhara region reported more than one SGP disease outbreaks in this time period. A total of 663 SGP disease outbreaks were reported in Eastern Amhara region during the study period. Most of these outbreaks were from South Wollo zone (37.56%), North Shoa (30.32%), and North Wollo (23.23%) (Table 5). The incidence of the SGP outbreak in the zones of the Eastern Amhara per seven district years is depicted in (Figure 3).

The average incidence of sheep and goat pox disease outbreaks at the district level was 8.61 over all 7 district years or 1.23 per district year. The lowest incidences were observed in the Wag Hemira zone and Oromia. Whereas the highest numbers of outbreaks were documented in the South Wollo zone and North Wollo zone (Table 5). The SGP outbreak incidence was above the average incidence of the region in South Wollo (10.82/7 district years) and North Wollo (10.26/7 district years) (Figure 3). There is a significant difference between all administrative zones of the study area (*p* < 0.001) with (*X*^2^ = 33.76) in the occurrence of sheep and goat pox disease outbreaks.

## 4. Discussion

Sheep and goat pox diseases are considered significant contagious viral diseases, which are highly widespread and found in the area of study, thus posing a serious problem. This is not only because of its wide distribution but also leading to death of both sexes and all ages of sheep and goats. This probably contributes to the extensive occurrence of SGP infections in this area as in endemic areas.

The study by Fentie et al. [17] reported that the highest outbreak was recorded in North Shoa (154) followed by South Wollo (115), and the least was recorded in Wag Hemira (12) in the period from January 2010 to December 2014. Whereas, in the current study, the highest outbreak was found in South Wollo (249) followed by North Shewa (201) and the least in Wag Hemira (23). Even if there is a difference in figures (i.e., number of outbreaks), both studies showed that South Wollo and North Shewa are highly affected while Wag Hemira is less affected. The differences may be due to the difference in the duration of the study, reporting each and every outbreak of sheep and goat pox disease by the districts and vaccination coverage difference between the study years and seasonality of the disease.

The numbers of SGP outbreaks reported in the current study were higher than the 548 outbreaks reported by Fentie et al. [17] in whole Amhara region. In the current study, from a total of 663 SGP disease outbreaks, there were 16,825 cases and 1,334 deaths in Eastern Amhara region. These findings were lower than the 22,888 cases and 2,477 deaths reported by Fentie et al. [17]. The variations in number of cases and deaths may be due to the difference in the area coverage, since the previous study incorporated the whole region, but the current study covers the eastern part of the region and difference in the application of strategies to limit spreading and management of infected animals.

In this study, there were significant variations in the occurrence of SGP outbreaks between zones, months, and years. This agrees with the study conducted by AU-IBAR [25], in the greater horn of Africa and Kenya. According to AU-IBAR [25], seasonal weather variations and livestock mobility cause stress and can compromise immune response of animals; livestock mobility favors the contact between infected and susceptible herds; the presence of naïve populations within an infected region is a major predisposing factor during epidemics; parasitism, bacterial infections, and unregulated trade are favoring conditions for emergence of SGP disease outbreaks. As studied by Zangana and Abdullah [26], poor-conditioned animals, overcrowding, poor feeding, and general mismanagement and abnormal uses of vaccination appeared to be the main cause of distribution and susceptibility to infection with the pox virus.

Based on the data reports between the time periods of 2013 and 2019, the number of SGP outbreaks that will occur in each year from 2020 to 2026 was forecasted if a better control method is not applied. The forecasted result suggests that high number of SGP outbreak will occur more than the available SGP disease outbreak annual data. The forecasted results of this study, therefore, will pull attention and help policy makers to focus on the unusual situations to decide whether any disease control involvement is required to stop the progress of the occurrence of the disease in the future.

According to the report of Masoud et al. [7], seasonality of the diseases enables the viruses to survive for several months in wet and cold weathers, by association with lambing season and transportation of animals for marketing. In some zones of Eastern Amhara region, there was little number of outbreaks of sheep and goat pox disease. This may be due to underreporting of outbreaks by their administrative kebeles or districts. Hence, the cumulative effect of these errors results in small number of outbreaks documented in Ethiopia. A study by Bayissa and Bereda [27] indicated that transport and communication were the two limiting factors in disease reporting in Ethiopia. These problems could result in irregular or absence of outbreak reports for some remote health posts. In Ethiopia, sheep, goat, and cattle for LSD are vaccinated by SGP vaccines for control and prevention of the Capripox virus. However, there have been frequent reports on the insufficient protection provided by the vaccine against lumpy skin disease virus in Ethiopia. According to Fentie et al. [17] suggestions, this failure of vaccination would likely be caused by poor vaccine handling where the availability of electricity is limited for keeping cold chain. In rare cases, since the vaccine is provided by the government itself, some unethical and negligent professionals may not use the vaccine whenever the vaccinating areas are very far from the clinic. This has its own contribution for the occurrence of the disease in the area.

The occurrence of SGP outbreaks in all months of the year agrees with the report of Atalla and Alzuheir [28] who reported that SGP outbreak has occurred in all months of the year in Palestine. In the current study, the highest outbreak was recorded during November while the lowest was at May. However, according to Atalla and Alzuheir [28], the highest number of outbreaks occurred in January, while the fewest outbreaks and the lowest incidence, mortality, and coverage occurred in August, September, and October. Data for various parameters were collected by month for the period from April 1996 to March 2000. Bhanuprakash et al. [9] also reported that SGP outbreak occurred in all months of the year, but the highest number of outbreaks and the greatest mortality occurred in March, while the fewest outbreaks and the lowest incidence and mortality occurred in August in India. The difference may be due to the difference in the management, weather condition, and immune status of breeds of sheep and goats in the countries.

## 5. Conclusion

Sheep and goat pox disease is extensively spread and established in Eastern Amhara region. It has been reported in all administrative zones of Eastern Amhara regional states during the years 2013–2019. The average incidence of SGP outbreaks at the district level was 8.61 over 7 years. Outbreaks were seasonal and occurred more often in months of dry cold season (spring) and rainy seasons which cause stress in animals. The results of the spatiotemporal analysis and the forecasted value for years may serve as a guide for the routine surveillance and control measures of SGP disease in the region and the country as a whole.

## Figures and Tables

**Table 1:** Sheep and goat population in the study zones.

Zone	Sheep population	Goat population	Total	Reference
South Wollo	1373184	826468	2199652	[20]
Wag Hemira	418022	216029	634051	[21]
Kemissie	102272	180405	2826677	[22]
North Wollo	796906	612440	1409346	[23]
North Shewa	1533366	987869	2521235	[24]

**Table 2:** Annual distribution of SGP disease in Eastern Amhara region from September 2013 to December 2019 and forecasted outbreaks.

Year (GC)	No. of SGP disease outbreaks	Percentage	Predicted outbreaks	*p* value	*X* ^2^
2013	29	4.37	—	0.000	50.65
2014	15	2.26	—		
2015	189	28.51	—		
2016	121	18.25	—		
2017	75	11.31	—		
2018	135	20.36	—		
2019	99	14.93	—		
2020			143		
2021			155		
2022			167		
2023			179		
2024			191		
2025			203		
2026			215		

**Table 3:** Monthly distribution of SGP disease in Eastern Amhara region from September 2013 to December 2019.

Months	No. of outbreaks	*p* value	*X* ^2^
September	73	0.000	78.28
October	77		
November	105		
December	48		
January	54		
February	43		
March	47		
April	44		
May	27		
June	37		
July	32		
August	76		
Total	663		
Average	55.25		

The morbidity rate was high during 2015, whereas the mortality and case fatality were high during 2016 (Table 4).

**Table 4:** Annual statistics of SGP outbreak in Eastern Amhara region from September 2013 to December 2019.

Year	No. of outbreaks	No. of deaths	No. of susceptible population	No. of cases	Morbidity rate (%)	Mortality rate (%)	Case fatality (%)
2013	29	69	180080	564	0.31	0.04	12.23
2014	15	17	54072	421	0.78	0.03	4.04
2015	189	524	993908	8210	0.82	0.05	6.38
2016	121	270	302532	1762	0.58	0.09	15.32
2017	75	95	238090	1124	0.47	0.04	8.45
2018	135	278	464714	3801	0.82	0.06	7.31
2019	99	81	398443	943	0.24	0.02	8.59
Total	663	1334		16825			

**Table 5:** Incidence and spatial distribution of SGP disease outbreaks in Eastern Amhara zones from September 2013 to December 2019.

Zones	No. of outbreaks	District number	Percentage	Incidence of SGP diseases outbreaks per district years
North Wollo	154	15	23.23	1.47
North Shewa	201	24	30.32	1.19
Oromia zone	36	7	5.43	0.73
South Wollo	249	23	37.56	1.54
Wag Hemira	23	8	3.46	0.41
Total	663	77	20	1.23

*p*=0.001 with *X*^2^ = 33.76.

## Data Availability

The data used to support the findings of this study are available from the corresponding author upon request.
